# A commentary on ‘Macrophage morphology and distribution are strong predictors of prognosis in resected colorectal liver metastases: results from an external retrospective observational study’

**DOI:** 10.1097/JS9.0000000000000466

**Published:** 2023-05-24

**Authors:** Yuan Chen, Chengcheng Wang, Qiang Xu

**Affiliations:** Department of General Surgery, State Key Laboratory of Complex Severe and Rare Diseases, Peking Union Medical College Hospital, Chinese Academy of Medical Sciences, Peking Union Medical College, Beijing, People’s Republic of China

*Dear Editor,*

We read with great interest the article entitled ‘Macrophage morphology and distribution are strong predictors of prognosis in resected colorectal liver metastases: results from an external retrospective observational study’ published in the recent issue of the *International Journal of Surgery* ^1^. The authors mainly demonstrated the role of tumor-associated macrophage (TAM) areas Large-TAMS or Small-TAMS (L-TAM or S-TAM) on colorectal liver metastases (CLM) in stratifying patient outcomes and predicting disease recurrence. However, despite the outstanding significance of the article, we still have some concerns about the conclusion.

First, for clinicopathological baseline statistics, the authors did not include indicators reflecting liver status as well as systemic inflammation. Since it has been reported that localized liver disease was able to modulate the morphology and function of hepatic macrophages^2^, it might contribute to the different basal macrophage infiltration and status in the CLM of patients in the analysis. And the systemic inflammatory status also determined the local macrophage infiltration and status in the liver to some extent^3,4^. Therefore, these points may be among the confounding factors that negatively affect the reliability of the conclusions.

Second, location heterogeneity might exist in the section-based calculation of TAM perimeter and area. Both the direction and position of the sections were able to affect the TAM perimeter and area to varying degrees. However, the authors did not mention the criteria for the selected sections, which might lead to a certain selection bias.

Third, the authors have now demonstrated the role of morphometric characterization of TAMs in CLM in predicting the prognosis of patients with colorectal cancer. On this basis, whether the morphometric characterization of TAMs in the primary site of the tumor possessed a similar role in predicting the prognosis of patients. Since not all patients progressed to CLM, the morphometric characterization of TAMs in the primary tumor site might be more universal.

Overall, we appreciate the authors’ efforts in reviewing the association between morphological features of TAM in CLM and patients’ outcomes, which provide a strong foundation for pathological prediction of prognosis in patients with colorectal cancer. However, more large-scale and rigorous cohorts still need to be applied to validate the clinical significance of TAM morphological features of CLM and primary tumors of colorectal cancer.

## Ethical approval

Not applicable.

## Consent

Not applicable.

## Sources of funding

This study was supported by the CAMS Innovation Fund for Medical Sciences (2021, 2021-1-I2M-002), the National Nature Science Foundation of China (2021, 82102810) and a fellowship of China Postdoctoral Science Foundation (2021, 2021M700501) (2022, 2022T150067) and National High-Level Hospital Clinical Research Funding (2022, 2022-PUMCH-D-001).

## Author contribution

Y.C.: study concept and writing; C.W. and Q.X.: critical revision of the manuscript for important intellectual content; C.W. and Q.X.: obtained funding.

## Conflicts of interest disclosure

No potential conflicts of interest were disclosed by the authors.

## Research registration unique identifying number (UIN)

Not applicable.

## Guarantor

Yuan Chen, Chengcheng Wang, and Qiang Xu.

## Provenance and peer review

Our paper was not invited.

## Data availability statement

Not applicable.
